# The Intersection of Autism and Gender Diversity in the Canadian Clinical Context: Characterizing a Sample of Adults Referred to Canada's Largest Publicly Funded Adult Gender-Care Service: Intersection de l’autisme et de la diversité des identités de genre dans le contexte clinique canadien : Caractérisation d’un échantillon d’adultes orientés vers le plus grand service de soins en matière d’identité de genre destinés aux adultes financé par le secteur public au Canada

**DOI:** 10.1177/07067437261442432

**Published:** 2026-04-24

**Authors:** Molly Isabel Pascoe, Jacqueline Vincent, Meng-Chuan Lai, Pushpal Desarkar, Nina Vitopoulos, Yona Lunsky, Kinnon Ross MacKinnon, June Sing Hong Lam

**Affiliations:** 1 Department of Psychology, Faculty of Arts and Science, 7938 University of Toronto, Toronto, Ontario, Canada; 2 Margaret and Wallace McCain Centre for Child, Youth & Family Mental Health, Campbell Family Mental Health Research Institute, 7978 Centre for Addiction and Mental Health, Toronto, Ontario, Canada; 3 Gender Identity Clinic, 7978 Centre for Addiction and Mental Health, Toronto, Ontario, Canada; 4 Azrieli Adult Neurodevelopmental Centre, Campbell Family Mental Health Research Institute, Centre for Addiction and Mental Health, Toronto, Ontario, Canada; 5 Department of Psychiatry, Temerty Faculty of Medicine, 7938 University of Toronto, Toronto, Ontario, Canada; 6 Department of Psychiatry, 7979 The Hospital for Sick Children, Toronto, Ontario, Canada; 7 Autism Research Centre, Department of Psychiatry, 2152 University of Cambridge, Cambridge, UK; 8 Department of Psychiatry, 38006 National Taiwan University Hospital and College of Medicine, Taipei, Taiwan; 9 Adult Neurodevelopmental Services and Azrieli Adult Neurodevelopmental Centre, 7978 Campbell Family Mental Health Research Institute, Centre for Addiction and Mental Health, Toronto, Ontario, Canada; 10 Department of Psychiatry, Temerty Faculty of Medicine, 7978 The University of Toronto, Toronto, Ontario, Canada; 11 Campbell Family Mental Health Research Institute, Centre for Addiction and Mental Health, Toronto, Ontario, Canada; 12 School of Social Work, 7991 York University, Toronto, Ontario, Canada; 13 Dalla Lana School of Public Health, 7938 University of Toronto, Toronto, Ontario, Canada; 14 Institute for Mental Health Policy Research, Campbell Family Mental Health Research Institute, Centre for Addiction and Mental Health, Toronto, Ontario, Canada

**Keywords:** autism, gender diversity, transgender, gender-related clinical care, autistic, adults

## Abstract

**Background:**

Research indicates a significant overlap between transgender and gender-diverse (TGD) and autistic identities. This intersectional population has higher risks of mental health challenges and worse mental health outcomes than individuals with just one of the two identities. Limited research focuses on adults at this intersection and their care access needs. To better characterize this population in the Canadian context, this study examines the population referred to Canada's largest publicly funded adult gender-related care clinic and compares demographic and diagnostic characteristics between those with and without a pre-existing autism diagnosis.

**Methods:**

The data come from the medical records of 1,843 adults referred to the Gender Identity Clinic (GIC) at the Centre for Addiction and Mental Health in Toronto, Canada, between January 2020 and March 2025. The prevalence of autism diagnosis prior to entering the clinic was calculated. Average age, sex-assigned-at-birth composition, prevalence of gender dysphoria diagnoses and of additional mental health and neurodevelopmental diagnoses were compared between autistic and non-autistic groups. Changes across time in the number of autistic individuals referred to the GIC were analyzed.

**Results:**

Approximately 6.3% of adults referred to GIC had a diagnosis of autism. The autistic and non-autistic groups had no difference in average age. The groups had no differences in sex-assigned-at-birth distribution. Autistic adults had greater rates of gender dysphoria. Autistic adults had higher rates of each category of mental health and neurodevelopmental diagnoses examined.

**Conclusions:**

This study is a first step in developing a holistic understanding of the experiences of autistic TGD adults seeking clinical gender-related care in the Canadian context, providing a starting point to addressing needs and barriers to care for this population, as well as insight into the substantial mental health challenges experienced by this population.

## Plain Language Summary Title

Characterizing the population of autistic adults visiting a large gender-care service in Canada

**Plain Language Summary:**

Many transgender and gender-diverse (TGD) individuals are autistic. They are more likely to have mental health problems but have a harder time getting mental health care. We wanted to know how common it was for the people coming to get gender care services at a large gender related clinic to have an autism diagnosis, and how those with an autism diagnosis were the same or different than other people at the clinic. We collected data from the medical records of 1,843 adults who had been referred to the Gender Identity Clinic at a large hospital in Toronto, Canada between January 2020 and March 2025. We examined the number of adults in the population who had a recorded diagnosis of autism at the point of intake, and compared the autistic and non-autistic groups in terms of: average age, sex-assigned-at-birth makeup, the rates of participants with a diagnosis of gender dysphoria, and other mental health or neurodevelopmental diagnoses. We also looked at the rates of adults with an autism diagnosis accessing care at the clinic across time. The researchers found that about 6.3% of the sample had a diagnosis of autism upon referral to the clinic. The autistic and non-autistic groups had no differences in average age. The sex-assigned-at-birth makeup of the two groups was equal. The autistic group was more likely to have higher rates of gender dysphoria and of mental health diagnoses than the non-autistic group. This study highlights the mental health difficulties experienced by this population, urging researchers, clinicians, families, and self-advocates to work toward removing the barriers experienced by this population in accessing care.

## Résumé

**Contexte:**

La recherche indique un chevauchement important entre l’identité transgenre et l’identité de genres multiples (TGD), d’une part, et les identités autistiques, d’autre part. La population occupant cette intersection présente des risques plus élevés de problèmes de santé mentale et subit des conséquences plus graves sur le plan de la santé mentale que les personnes ne présentant qu’une seule de ces deux identités. Peu de projets de recherche sont menés sur les besoins en matière d’accès aux soins des adultes occupant cette intersection. Afin de mieux caractériser cette population dans le contexte canadien, cette étude évalue la population orientée vers la plus grande clinique de soins liés aux identités de genre destinés aux adultes financée par le secteur public au Canada. De plus, elle compare les caractéristiques démographiques et diagnostiques des personnes ayant reçu un diagnostic d’autisme préexistant à celles n’en ayant pas.

**Méthodes:**

Les données proviennent des dossiers médicaux de 1 843 adultes orientés vers la Clinique de l’identité du genre (CIG) du Centre de toxicomanie et de santé mentale (CAMH) de Toronto, au Canada, de janvier 2020 à mars 2025. La prévalence du diagnostic d’autisme avant l’arrivée à la clinique a été calculée. L’âge moyen, la répartition du sexe attribué à la naissance, la prévalence des diagnostics de dysphorie de genre, d’autres problèmes de santé mentale et de troubles neurodéveloppementaux dans les groupes de personnes autistes et non autistes ont fait l’objet de comparaisons. L’évolution du nombre de personnes autistes orientées vers le GIC au fil du temps a été analysée.

**Résultats:**

Environ 6,3% des adultes orientés vers le GIC avaient un diagnostic d’autisme préexistant. Les groupes de personnes autistes et non autistes ne présentaient aucune différence pour ce qui est de l’âge moyen. Les groupes ne présentaient aucune différence au chapitre de la répartition du sexe attribué à la naissance. Les adultes autistes présentaient des taux plus élevés de dysphorie de genre. Les adultes autistes présentaient des taux plus élevés de chacune des catégories diagnostiques de problèmes de santé mentale et de troubles neurodéveloppementaux évaluées.

**Conclusions:**

Cette étude constitue la première étape de la compréhension holistique des expériences des adultes autistes ayant une identité transgenre et une identité de genres multiples qui cherchent à obtenir des soins cliniques liés à l’identité de genre au Canada. Cela fournit un point de départ pour répondre aux besoins et surmonter les obstacles en matière de soins offerts à cette population, ainsi qu’un aperçu des problèmes de santé mentale importants auxquels fait face cette population.

Recent research shows a clear and significant overlap between transgender and gender-diverse (TGD) and autistic populations,^1,2^ with the prevalence of autism among TGD individuals recently estimated at 11%.^3^ TGD people make up approximately 0.33% of the general Canadian population.^4^ A recent review of global data shows a consistent increase over time in the proportion of the population who identify as TGD.^5^ Autism is one of the most common neurodevelopmental diagnoses, diagnosed for approximately 1 in 50 (approximately 2%) of children and youth in Canada^6^ and 1% of adults.^7^

It is well recognized that autistic adults have higher rates of mental health diagnoses than their neurotypical peers,^8,9^ and greater difficulties accessing mental health services than non-autistic individuals.^a,10,11^ TGD adults similarly experience higher rates of mental health concerns^12,13^ and greater difficulties accessing appropriate mental health services^14^ than their cisgender peers. There is, however, limited research available on the mental health outcomes of adults who are both autistic and gender-diverse; the existing literature indicates a higher risk of depression and anxiety for autistic TGD individuals compared to peers who are either TGD and non-autistic or cisgender and autistic,^15^ along with higher rates of self-harm and suicidal ideation.^16^ Previous theorists have extended the minority stress model, a conceptualization of the stress and negative health outcomes experienced by minoritized groups,^17^ to TGD^18^ and autistic populations.^19^ Autistic TGD individuals have at least doubled minority status and thus experience both types of minority stress, along with unique stressors associated with the intersectionality of these identities.^20^ This is exemplified in Kung's finding that both autistic traits and gender minority stress correlate with worse overall mental health outcomes in TGD adults.^21^

There is a clear need for tailored health care for autistic TGD people^22^; however, issues of long waitlists, regionalized subspecialty care, and barriers to services have been identified by both TGD^23^ and autistic adults,^24,25^ reflecting two marginalized populations with unique, and often unmet, needs. Adults at the autism-TGD intersection report that it is already challenging to seek either gender-affirming care or services for autistic adults. They experience additional frustration and fatigue associated with a lack of systems and care providers who adequately understand the experiences and needs of those seeking support for both conditions.^26,27^ These services are typically siloed, and while there is indication of an overrepresentation of autism in adults seeking gender-affirming care in some jurisdictions,^28^ there are very few clinical services that specifically serve people who are *both* autistic and gender-diverse. This is despite previous research suggesting the importance of specialized care.^29,30^ Much is still to be understood through research to identify the service needs of this underserved population and to better strategize on meeting those needs.

As a starting point, the current study characterizes the clinical population referred to Canada's largest publicly funded adult gender-related clinical care service^31^ at the Centre for Addiction and Mental Health (CAMH), a large, tertiary mental health hospital in Toronto, Ontario, Canada. Two previous quantitative studies have looked at the intersection of autism and gender diversity in Canadian samples. Adams and colleagues identified group differences in demographic variables between autistic and non-autistic TGD individuals participating in a Canadian national survey on TGD health.^32^ Their community-based study found that autistic adults were, on average, younger than non-autistic adults, that there were no significant differences between groups in terms of sex-assigned-at-birth makeup, and that autistic adults experienced greater mental health challenges along with unmet mental health and healthcare needs. Mo and colleagues examined gender diversity across neurodivergent and neurotypical children and adolescents in Canada.^33^ They examined participants with or without neurodevelopmental conditions and found that higher levels of autistic traits, but not categorical autism or attention-deficit/hyperactivity disorder (ADHD) diagnoses, correlate with higher scores on a dimensional measure of gender diversity. The present study builds on the work of Adams and colleagues^32^ Mo and colleagues^33^ by adding insight specifically into the experiences of a clinical adult population. The present study is the first of its kind to specifically examine the profile of autistic versus non-autistic adults accessing clinical gender-related care in Canada. Identifying the characteristics of this clinical sample also sheds light on who is currently able to access care such that future research and program planning can work toward removing service barriers and offering tailored care.

We hypothesized that autistic individuals would make up around 11% of this clinical sample, based on the meta-analytic findings of Kallitsounaki and Williams.^3^ We examined differences in age between the autistic and non-autistic groups, as well as differences in sex-assigned-at-birth composition between groups. We hypothesized that the autistic group would be younger than the non-autistic group, based on the findings of Adams and colleagues.^32^ Our analyses on sex-assigned-at-birth differences were exploratory, given the dearth of previous research on adults, though we kept in mind findings on adolescent populations of higher rates of assigned-female-at-birth (AFAB) individuals with autism diagnoses accessing gender-related clinical care.^34^ Additionally, we explored potential differences between groups in diagnosed gender dysphoria. We also examined differences between groups in mental health diagnoses, and hypothesized that the autistic group would have overall a greater number of mental health diagnoses within each diagnostic category and across categories.^9,15,21^

## Methods

### Data Source

Data for this study came from chart records of 1,843 adults (aged 18 years or older) referred to the Adult Gender Identity Clinic (GIC) at CAMH in Toronto, Ontario, Canada between January 2020 and March 2025. Table 1 includes relevant descriptive statistics on demographic and clinical characteristics of the sample. The GIC is a multidisciplinary public healthcare service providing assessments and gender-affirming care to TGD individuals referred by their primary care providers, predominantly within Ontario.^31^ Demographic and clinical characteristics were extracted from the electronic medical record (EMR). The CAMH Research Ethics Board reviewed and approved the study (REB # 2023/013). Adults were coded in a binary nature as autistic or non-autistic according to whether or not the individual had received a diagnosis of autism prior to being referred to the clinic, with 0 = non-autistic and 1 = autistic. All adults with a recorded diagnosis of autism were listed as autistic, including those with other neurodevelopmental diagnoses as well (e.g., ADHD). Sex-assigned-at-birth was coded as 0 = assigned-male-at-birth (AMAB), 1 = AFAB, 2 = intersex, 3 = unknown/other. Adults were coded in a binary nature in terms of whether or not they had received a diagnosis of gender dysphoria after assessment at the clinic, with 0 = does not have a gender dysphoria diagnosis and 1 = has a gender dysphoria diagnosis. Mental health diagnoses were split into the following categories: mood disorders, anxiety disorders, obsessive-compulsive and related disorders, trauma-related disorders, substance use disorders, psychotic disorders, personality disorders, other mental health diagnoses (e.g., eating and feeding disorders, dissociative disorders, somatic symptom and related disorders, etc.), and neurodevelopmental diagnoses other than autism (including learning disability, intellectual disability, and ADHD). Data were coded in a binary format, and participants were coded as 1 if they had at least one diagnosis in that category and as 0 if they did not.

**Table 1.** Demographic and Clinical Characteristics.

	Autistic group	Non-autistic group	Group comparison statistics
Age (in years)	*M* = 25.66 *SD* = 7.34	*M* = 27.61 *SD* = 10.13	U = 107786 *P* = .168 *r* = 0.032
Sex-assigned-at-birth	AFAB: 47.4% AMAB: 50.9% Intersex: 0% Unknown/Other: 1.7%	AFAB: 48.5% AMAB: 45.9% Intersex: 1.1% Unknown/Other: 4.9%	F-F-H test: *P* = .310 *V* = 0.048 *X*^2^ test: *X*^2^ = .270 *P* = .603 *V* = 0.015
Gender dysphoria diagnosis	87.1%	75.0%	*X*^2^ = 7.99 *P* = .005 *V* = 0.068
Mood disorder diagnosis	38.8%	11.8%	*X*^2^ = 20.42 *P* < .001 *V* = 0.137
Anxiety disorder diagnosis	33.3%	11.0%	*X*^2^ = 65.43 *P* < .001 *V* = 0.192
Obsessive-compulsive and related disorder diagnosis	10.3%	1.16%	*P* < .001 *V* = 0.171
Trauma-related disorder diagnosis	21.6%	5.4%	*X*^2^ = 44.07 *P* < .001 *V* = 0.159
Substance use disorder diagnosis	21.6%	7.1%	*X*^2^ = 29.14 *P* < .001 *V* = 0.130
Personality disorder diagnosis	19.8%	7.0%	*X*^2^ = 23.06 *P* < .001 *V* = 0.116
Psychotic disorder diagnosis	6.0%	1.9%	*P* = .009 *V* = 0.071
Other mental health disorder diagnosis	10.3%	1.0%	*P* < .001 *V* = 0.179
Neurodevelopmental diagnoses other than autism	42.2%	5.84%	*X*^2^ = 187.72 *P* < .001 *V* = 0.323
Total mental health diagnoses	Mdn = 2 IQR = 2	Mdn = 0 IQR = 0	U = 37720 *P* < .001 *r* = −0.338
*n*	116	1727	

*Note:* “M” refers to mean, “SD” refers to standard deviation. “Mdn” refers to median, “IQR” refers to interquartile range. “*X*^2^” refers to chi-square, “*P*” refers to *P*-value, “*V*” refers to Cramér's V, “*r*” refers to rank-biserial correlation. “F–F–H” refers to the Fisher–Freeman–Halton Test. “AFAB” refers to those assigned female at birth, “AMAB” refers to those assigned male at birth. “*N*” refers to the number of participants.

### Analyses

Descriptive statistics and frequencies were calculated for all relevant variables. A Mann–Whitney *U* test was performed to assess for mean age differences between the autistic and non-autistic groups. A Fisher-Freeman-Halton test was done to test for differences in the frequency of participants of each sex-assigned-at-birth between autistic and non-autistic groups. A chi-squared test was done to assess for differences in AMAB versus AFAB makeup of the autistic and non-autistic groups, given the low sample sizes of the other sex-assigned-at-birth groups (intersex and unknown/other) and the acknowledged conservativeness of Fisher–Freeman–Halton tests (i.e., it has the tendency to result in higher *P*-values).^35^ An additional chi-squared test was done to assess for differences in the frequency of adults with a gender dysphoria diagnosis between the autistic and non-autistic groups. Chi-squared tests were done to test for differences in rates of mental health diagnoses for each diagnostic category between autistic and non-autistic groups. Fisher's exact tests were used for three diagnostic categories (obsessive-compulsive and related disorders, psychotic disorders, and other mental health diagnoses) for which expected cell sizes did not meet the requirements for performing a chi-squared test. Total diagnoses across categories were tallied, and a Mann–Whitney *U* test was performed, looking at differences in the number of diagnoses between the autistic and non-autistic groups.

The required assumption checks were performed prior to running each test. Statistical tests were conducted in R version 4.4.1^36^ using the base package.^36^ Complete case analysis was used to handle missing responses.

## Results

Table 1 presents frequency calculations, descriptive statistics, test statistics, and effect sizes for all variables. Unless otherwise indicated, all assumptions for statistical tests were met. Approximately six percent (6.3%) of adults referred to GIC had a clinical diagnosis of autism recorded in their EMR.

The participant age variable did not meet the assumptions of homoscedasticity and normality, so a Mann–Whitney *U* test was used to look at group differences between the autistic and non-autistic groups. There were no significant differences (*P* =.168) between the autistic (*M* = 25.66, *SD* = 7.34) and non-autistic groups (*M* = 27.61, *SD* *=* 10.13).

The Fisher–Freeman–Halton test examining the frequency of sex-assigned-at-birth between the autistic and non-autistic groups yielded non-significant results, *P* = .310. The chi-squared test comparing AMAB versus AFAB composition of the two groups yielded non-significant results, *P* = .222, indicating no differences between groups in terms of sex-assigned-at-birth.

Approximately 75.7% of all adults referred to GIC were diagnosed with gender dysphoria after assessment at the clinic. There was a small but significant difference in prevalence between the autistic and non-autistic groups, with a greater frequency of adults experiencing gender dysphoria in the autistic group compared to the non-autistic group, *P* = .005.

A significantly greater frequency of adults had a mood disorder in the autistic group as compared to the non-autistic group, *P* < .001. A significantly greater frequency of adults had an anxiety disorder in the autistic group as compared to the non-autistic group, *P* < .001. A significantly greater frequency of adults had an obsessive-compulsive and related disorder in the autistic group as compared to the non-autistic group, *P* < .001. A significantly greater frequency of adults had a trauma-related disorder in the autistic group as compared to the non-autistic group, *P* < .001. A significantly greater frequency of adults had a substance use disorder in the autistic group as compared to the non-autistic group, *P* < .001. A significantly greater frequency of adults had a personality disorder diagnosis in the autistic group as compared to the non-autistic group, *P* < .001. A significantly greater frequency of adults had a psychotic disorder in the autistic group as compared to the non-autistic group, *P* = .009. A significantly greater frequency of adults had a mental health diagnosis classified as other in the autistic group as compared to the non-autistic group, *P* < .001. A significantly greater frequency of adults had a neurodevelopmental condition other than autism in the autistic group as compared to the non-autistic group, *P* < .001. Finally, we compared the number of mental health conditions across categories between the autistic and non-autistic groups. Given that the data did not meet the assumptions of normality or homoscedasticity, a Mann–Whitney *U* test was performed. A significantly greater number of mental health diagnoses were found in the autistic group, as compared to the non-autistic group, *P* < .001.

## Discussion

This first study of the prevalence of autism and associated demographic and clinical features in a Canadian gender-diverse clinical sample gives us insight into the profile of the autism-TGD intersection in a Canadian clinical context. Our study revealed that 6.3% of adults referred to the GIC had a clinical diagnosis of autism recorded before being seen at the clinic. This exceeds the prevalence of autism in the global adult population (i.e., 1%),^9^ but is lower than we originally hypothesized based on the meta-analysis-derived estimates (11%) from Kallitsounaki and Williams.^3^ A notable difference is that the present study examined specifically a sample of adults referred for clinical gender-related care, whereas Kallitsounaki and Williams’ meta-analysis summarizes studies of those seeking clinical care and general population-based studies, and general population studies are often based on self-report so may include self-identifying autistic individuals who do not necessarily have a diagnosis, unlike the present study.^3^

Autistic individuals have more challenges accessing gender-related clinical care than nonautistic individuals due to executive functioning- and communication-related barriers,^37^ and bureaucratic and economic barriers.^26^ Further, autistic adults report that many clinicians lack knowledge on autistic TGD experiences^26^ and discount autistic experiences of gender diversity^38^ and autistic health care needs more generally.^39^ With these barriers in mind, it follows that a sample from a gender-related care clinic would include fewer autistic individuals than a general population sample. It remains difficult to access an assessment for autism for the general Canadian population,^40^ but likely especially so for TGD people who often experience additional barriers, including greater socioeconomic marginalization.^41,42^ Our estimate of the percentage of the sample who were autistic may be lower due to the inaccessibility of receiving an autism diagnosis. We note that many individuals are diagnosed with autism at a later time at the GIC and/or are referred to specialized adult autism services at CAMH. Additionally, prior research suggests that autistic TGD patients will minimize or pre-emptively avoid sharing information about autism during transition-related readiness assessments.^32^ Thus, we expect that 6.3% is an under-estimate of the prevalence of autism in this clinical population. We view this estimate as a baseline, and encourage future researchers to replicate our study using a more inclusive method of defining who is and is not autistic.

We found no significant differences in average age between the autistic and non-autistic groups. Unlike the present study, Adams and colleagues’ recent cross-sectional national survey found that autistic TGD Canadians were significantly younger than non-autistic TGD Canadians.^32^ One explanation as to why our findings differ from those of Adams and colleagues^32^ may have to do with the reported cohort effects in autism diagnoses.^43^ Historically, AFAB individuals have been much less likely to receive an autism diagnosis than AMAB individuals,^44^ because autism diagnoses and traits were more likely to be missed in AFAB individuals,^45^ given differences in trait presentation.^46,47^ Given the increase in awareness over time of the way that autism presents in AFAB individuals,^47,48^ there are cohort effects in the rates of autism being diagnosed in AFAB individuals,^43^ with younger AFAB adults potentially being more likely to have received an autism diagnosis. We note that Adams and colleagues (2024) had a lower proportion of AMAB-to-AFAB individuals in their sample overall (0.57:1), as compared to our sample (1:1). Given the higher rates of AFAB individuals in the study by Adams and colleagues,^32^ these cohort effects may partially explain their finding of lower age in the autistic group.

We found no significant differences between groups in sex-assigned-at-birth composition. Kahn and colleagues’ study on the EMRs of adolescents accessing gender-related clinical care in the United States found higher rates of AFAB adolescents in their sample.^34^ The difference between our findings and those of Kahn and colleagues^34^ could also be due to the increased recognition over time of how autism presents in AFAB individuals^47,48^ and associated cohort effects.^43^ There is a higher proportion of AMAB-to-AFAB individuals presenting to the GIC overall, compared to community and other samples,^32^ which may reflect the requirement for a second assessor for AMAB individuals to be eligible for government funding for vaginoplasty. However, the difference in the sex-assigned-at-birth proportion may also reflect the makeup of TGD individuals with greater mental health complexity that primary care providers felt required a referral to the GIC to support their transition.

Further, we found a small but significant difference between autistic and non-autistic groups in gender dysphoria diagnosis rates. Strang and colleagues note that autistic individuals often experience excess scrutiny from clinicians regarding their experiences of gender diversity, beyond that which is typically experienced by the general population in assessments.^38^ Our finding of greater diagnosed gender dysphoria in the autistic group can serve as a reminder for clinicians that autistic individuals seeking gender-related clinical care are even more likely to experience gender-related distress than non-autistic individuals, and should be able to access care accordingly.

Regarding differences in the frequency of mental health diagnoses between autistic and non-autistic groups, we found that these gender-referred autistic adults had between three and nine times the rates of each category of mental health and neurodevelopmental diagnoses as compared to the gender-referred non-autistic adults. We also found that, overall, autistic individuals were more likely to have a greater number of mental health diagnoses across categories as compared to the non-autistic group. This fits in with previous literature findings of more mental health needs and worse mental health outcomes in individuals who are autistic and TGD,^49^ as opposed to individuals who are non-autistic and TGD, and highlights the necessity to alleviate barriers faced by this population in accessing mental health care. This also fits with prior literature on mental health differences between autistic and non-autistic populations more generally.^50^ High rates of substance use in the autistic group may reflect the use of substances as a coping strategy in a neuro-normative world, along with the associated mental health challenges.^51,52^ This further emphasizes the need for adequate mental health and physical healthcare for autistic populations to promote well-being. The high rates of additional diagnoses in the autistic group may also reflect the common experience of receiving multiple diagnoses prior to receiving an autism diagnosis,^53^ particularly of other neurodevelopmental conditions, personality disorders, and psychotic disorders.^54^ Although all diagnoses were documented by a physician or nurse practitioner at the time of referral and are therefore clinician-verified rather than patient self-reported, chart-based diagnoses may not reflect the results of a structured diagnostic interview or formal assessment and could be subject to documentation variability. We also note that, due to the nature of CAMH's EMR system and the evolving nature of diagnoses over time, we opted to dichotomously report whether each individual had at least one diagnosis within each category, providing us with a less nuanced understanding of the magnitude of mental health problems.

With the aforementioned results in mind, we note some limitations in the methodology used in the present study, as well as future research directions. Firstly, we acknowledge a limitation in the categorical nature of how we define one being autistic, i.e., with an autism diagnosis. Measuring autism solely in terms of a categorical diagnosis prior to entering the clinic does not account for additional cases wherein a client is likely to have undiagnosed autism or to receive an autism diagnosis following first being seen by the clinic. Recent research recognizes the utility of using a dimensional approach in relevant contexts, given that quantitative scores of autistic traits may be more likely to show stronger correlations with other traits.^55^ This is exemplified in Kallitsounaki and Williams’ finding of a higher prevalence of autistic traits in gender-diverse cohorts as compared to the prevalence of categorical autism diagnoses.^3^ Further, using dimensional autism trait measures may be preferable in studies like the present one, given the vast heterogeneity in the expression of autistic traits^56^ and diagnostic disparities in minoritized groups.^57,58^ Nevertheless, we were bound by which data were included in the charts of those accessing services at the GIC, and as such, were only able to use a categorical approach in our definition of who is autistic. We recommend that future research replicate all analyses included in the present study with dimensional metrics of autistic traits.

Likewise, the presence of gender dysphoria was also denoted categorically, which likely impacted the findings of the analyses that included gender dysphoria as a variable. Accordingly, relevant areas for future research would be the better characterization of gender dysphoria in autistic populations. This future work could be especially informed by qualitative findings of unique experiences that likely differ in part from those of non-autistic populations with gender dysphoria.^59,60^

## Conclusion

This study examines prevalence rates and factors associated with the intersection of TGD identity and autism in a clinical sample of adults seeking gender-related care. It provides a starting point for building a holistic understanding of the experiences of autistic individuals seeking gender-related clinical care in Canada. Further, it highlights the urgency of improving access to mental health and gender-related clinical care services for this population, given the significant mental health needs. This preliminary investigation can be built upon to further the evidence base upon which to improve Canadian health services, and address the care needs of those at the intersection of autism and gender diversity.

## Notes

a. The research team would like to acknowledge that we decided to use "non-autistic" in describing the comparison group throughout this article for consistency, but we acknowledge that other language for this group exists, including "allistic." We also note that the comparison group likely includes autistic individuals who were not identified at the time of referral, so it is unlikely to be a fully allistic group.
